# Prognosis in home dental bleaching: a systematic review

**DOI:** 10.1007/s00784-023-05069-0

**Published:** 2023-06-05

**Authors:** Rossella Fioresta, María Melo, Leopoldo Forner, José Luis Sanz

**Affiliations:** Departament of Stomatology, Facultat de Medicina i Odontologia, Universitat de València, C. Gasgó Oliag, 1, 46010 Valencia, Spain

**Keywords:** Home dental bleaching, Home dental whitening, Prognosis, Systematic review, Clinical trials

## Abstract

**Objectives:**

The aim of this systematic review was to evaluate the prognosis of at-home dental bleaching using low concentration bleaching products.

**Materials and methods:**

This review was conducted was performed following the recommendations of the 2020 PRISMA statement and was registered in the International Prospective Register of Systematic Reviews (PROSPERO-CRD42022360530). The PICO question was “What is the prognosis of home teeth whitening treatment?”. An advanced electronic search was made in three databases: PubMed, Web of Science, and Embase.

**Results:**

The database search led to the retrieval of 225 articles. After elimination of duplicate references, the titles and abstracts of the articles were analyzed with respect to the eligibility criteria, and 24 studies were included for the development of the systematic review.

**Conclusions:**

Most authors state that the color remains stable between 1 and 2.5 years regardless of the type of bleaching agent or the forms of administration, and color stability in cases of severe discolorations presents a higher degree of recurrence.

**Clinical relevance:**

Given the growing demand for dental cosmetic treatments, the following systematic review may aid the clinician’s continuing education and evidence-based practice by providing knowledge on the field of at-home dental bleaching agents and their long-term effects.

**Supplementary Information:**

The online version contains supplementary material available at 10.1007/s00784-023-05069-0.

## Background

Nowadays, esthetic procedures are widely required in daily dental practice. Patients seeking an attractive smile have been increasing significantly, especially in terms of tooth color [1]. Dental bleaching is considered the treatment of choice to improve patient satisfaction in terms of tooth discoloration, due to its non-invasive approach and low cost compared to other cosmetic dentistry procedures [2]. Many types of color problems can affect the appearance of teeth, and the causes of tooth discoloration must be carefully evaluated to establish a correct diagnosis [3, 4].

Discolorations are classified as either extrinsic or intrinsic. Extrinsic discolorations result from the accumulation of chromogenic substances on the external surface of the tooth [3–5]. They are secondary to the habitual intake of chromogenic dietary sources such as wine, coffee, tea, carrots, oranges, chocolate, tobacco, some mouthwashes, or poor or incorrect oral hygiene habits. These discolorations, most of the times, can be eliminated mechanically by professional prophylaxis treatments [3–7].

As for intrinsic discolorations, they occur after a change in the structural composition or thickness of the dental tissues. They can be caused by systemic or local factors. Systemic causes include those related to drugs (e.g., tetracycline), alterations in the structure or thickness of dental tissues [5, 7]. Local causes include pulp necrosis, intrapulpal hemorrhage, remnants of pulp tissue in the chamber after root canal treatment, root canal filling materials, some coronal restorative materials, enamel microcracks, caries, and aging. These discolorations are treated by tooth bleaching techniques [3–6, 8]. Historically, bleaching techniques were introduced to the clinic in 1848 as a treatment for discolorations of non-vital teeth, using chloride of lime [9]. In 1864, Truman introduced a more effective technique for bleaching non-vital teeth that consisted in the application of a solution formed by chlorine and acetic acid [10].

At the end of the nineteenth century, other bleaching agents such as potassium cyanide, oxalic acid, sulfurous acid, aluminum chloride, sodium hypophosphate, pyrozone, hydrogen dioxide, and sodium peroxide were also successfully used in bleaching non-vital teeth [11–16]. Finally, in 1868, whitening techniques for vital teeth were introduced, using oxalic acid, pyrozone, and later hydrogen peroxide (HP) [11–16]. Dental bleaching techniques can be classified into vital and non-vital bleaching techniques. The former are classified as: in-office bleaching, at-home bleaching supervised by the dentist and at-home bleaching without supervision, using over-the-counter bleaching products [17].

Bleaching in the clinic uses bleaching products in high concentrations (25–40% HP). This procedure is carried out after having performed some previous maneuvers such as hard tissue prophylaxis and soft tissue protection through the application of physical barriers. The agents applied can be activated chemically or by light [18]. Alternatively, at-home dental bleaching involves the use of bleaching products with lower concentrations [5]. Originally, these techniques consisted of the application of 10% carbamide peroxide (CP) in individualized splints applied overnight (6–8 h). Currently, gels with concentrations up to 20% are applied [1, 19, 20].However, the industry has developed products that act more quickly compared to those described above and are more attractive to some patients. These products are presented in the form of gels containing HP in concentrations between 3 and 10%, mainly applied during the day [21, 22].

Supervision by the dentist takes place during check-up appointments [1, 19–23]. The advantages of this technique include: self-administration by the patient, less time in the dental chair, high degree of safety, fewer adverse effects, and low cost [24]. The disadvantages include the need for high patient collaboration since the result is linked to the diligence with which the indicated guidelines are respected. Excessive or prolonged use of the treatment may cause increased tooth sensitivity and soft tissue irritation. Sometimes both techniques can be combined [11, 24].

The third technique, also known as “over-the-counter,” involves the use of over-the-counter products containing low concentration bleaching agents. These products are purchased and applied without professional supervision and come in the form of toothpaste, whitening strips, mouthwashes, and prefabricated splints [5, 25].

The literature describes a wide variety of protocols and methods for applying the products described above, although there is still no single accepted protocol [23]. As for the stability of the color obtained after bleaching, this depends to a large extent on diet and habits as they contribute to the development of extrinsic discolorations [5].

The literature on the effectiveness of home bleaching is abundant and has already been reviewed [26, 27]. The term “effectiveness” refers to the ability of dental bleaching to produce an effect (color change). Alternatively, the term “prognosis” defines the stability and duration of the obtained effect, maintained over time. A series of studies on the prognosis of home dental bleaching have been performed. However, no efforts have been made to assess such evidence. Thus, the aim of this systematic review is to perform a qualitative synthesis of available studies on the prognosis of home dental bleaching using low-concentration products on vital teeth, in terms of duration of the effects achieved after being exposed to the treatment.

## Materials and methods

### Protocol and registration

This study protocol was registered in the International Prospective Register of Systematic Reviews (PROSPERO-CRD42022360530).

### Search strategy

This systematic review of the literature was performed following the recommendations of the PRISMA statement updated in 2020 [28]. A literature search was conducted in digital databases PubMed, Web of Science, and Embase in May 2022 and was updated on the 29th of March 2023. No language or time restrictions were applied. The search strategy employed three main fields: field #1, keywords regarding prognosis (prognosis or dura*); field #2, keywords regarding dental bleaching (“tooth bleaching” or “teeth bleaching” or “dental bleaching” or “dental whitening” or bleaching or whitening); and field #3, keywords regarding at-home dental bleaching specifically (home). Keyword selection was based on the descriptors used in previous studies in the field. Whenever possible, both controlled and uncontrolled terms were used. See Supplementary Table 1 for the specific search strategy for each database. Restrictions to publication date and language were not applied. The PICO question guiding the search was: What is the prognosis of home dental bleaching treatment? Which can be subdivided as follows:

P (population): individuals undergoing home dental bleaching; I (intervention): home bleaching using specific low concentration bleaching agent/s with specific application method/s; C (comparison): home bleaching using other specific low concentration bleaching agent/s and/or other specific application method/s; and O (outcomes).

The search strategy, study selection process, data extraction, and quality assessment (risk of bias assessment) were performed by two independent investigators (R.F. and M.M). In case of doubt, a third investigator was consulted (JL.S).

### Eligibility criteria

Studies were selected based on previously established inclusion criteria: (1) RCT; (2) clinical trials; (3) home bleaching protocols using HP in concentrations lower than 10% or CP in concentrations lower than 28%, with post-treatment follow-up times of no less than 2 months; (4) in vivo studies in patients of any age group. Studies in which in-office bleaching protocols were applied with concentrations not dispensable for home use, combined bleaching techniques, home VS in-office bleaching techniques, retrospective studies, in vitro, cohort, clinical cases, and case series were excluded.

### Study selection, data extraction, and synthesis of the evidence

All the selected articles were imported into a citation management software (Mendeley. Elsevier, Amsterdam, Netherlands), and duplicate articles were eliminated. A first screening of the articles according to title and abstract was then performed according to the search strategy described above, and finally a second screening of the full text of the eligible studies was performed.

The following bibliometric data were extracted from each study: author and year of publication. As for the methodological variables, the following were extracted: study type, diagnostic method, number of participants and dropout, bleaching protocol, bleaching product application method, age range, and bleaching method. Lastly, the following outcome variables were extracted: results in terms of DSGU/ΔE; results in terms of color change in ΔE values (if spectrophotometer was used); and results in terms of color change in DSGU (shade guide unit) values (if a shade guide was used).

After data extraction, a synthesis of the evidence was performed. To do so, the extracted variables were assessed to search for similarities and/or contradictions among the included studies’ results. Lastly, a qualitative synthesis of the studies’ data regarding the primary outcomes of this review (prognosis of home bleaching, measured over time by analysis of ΔE and color change in DSGU dental units) was performed.

### Quality assessment

Quality assessment of the selected studies was carried out using Cochrane Collaboration tools for risk of bias: ROB-2 for randomized controlled trials (RCTs) and ROBINS-I for controlled trials (CTs) [29]. No other analyses could be performed due to the heterogeneity of the data.

## Results

### Study selection

**Fig. 1:**
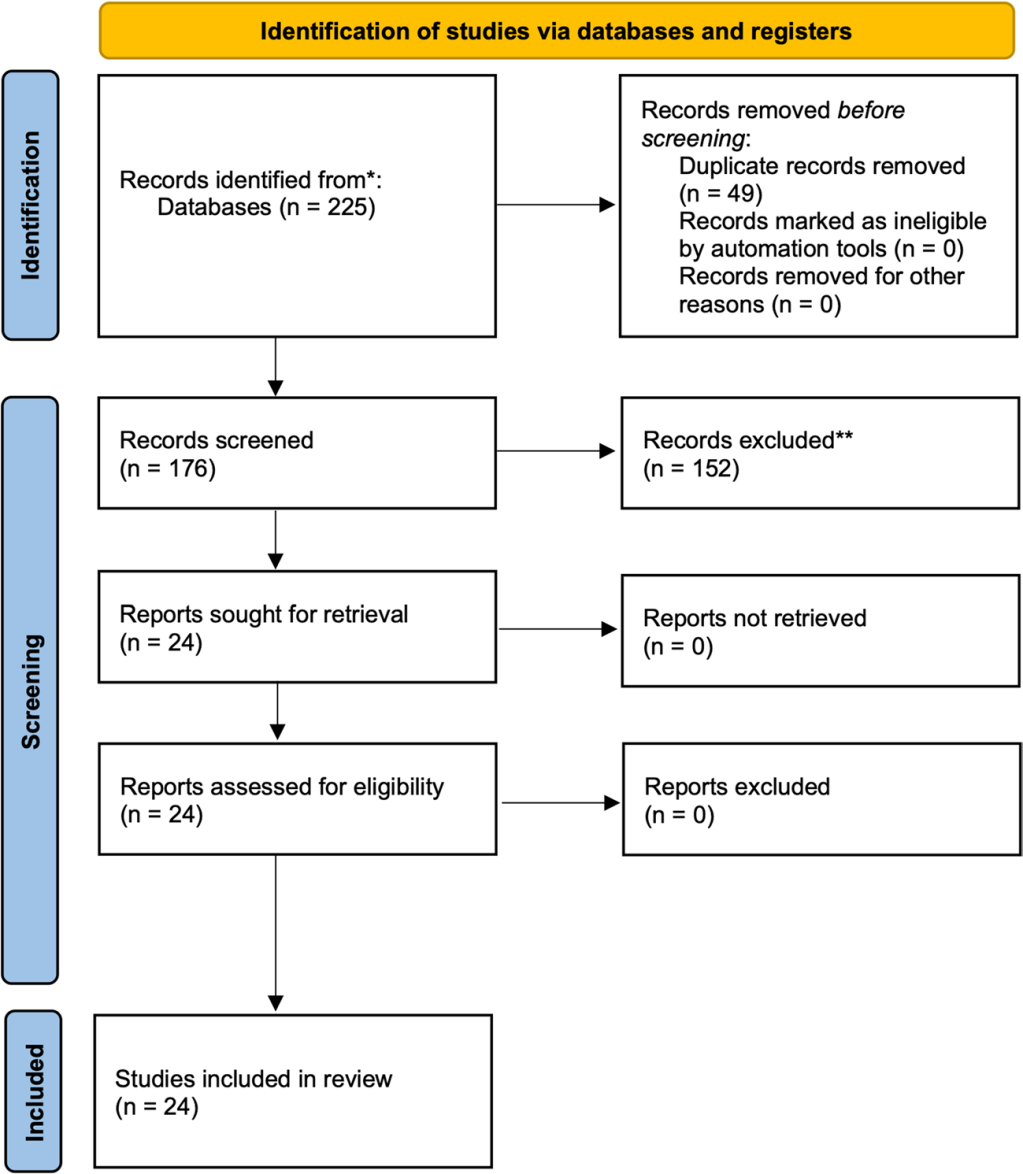
Study selection flow diagram

The database search led to the retrieval of 225 articles: 33 in Web of Science, 138 in PubMed, and 54 in Embase. After elimination of duplicate references, the titles and abstracts of the articles were analyzed with respect to the eligibility criteria, and 24 articles were selected for full-text reading. All of the assessed studies were eligible for the qualitative synthesis (Fig. 1).

### Study methodology

The characteristics of the 24 selected studies are described in Table 1 (methodology) and Table 2 (results). Sixteen randomized clinical trials [1, 2, 19–23, 30–38] and 8 clinical trials [39–46] were selected. In total, 1197 subjects were exposed to home bleaching using different bleaching agents such as HP at concentrations of 3% [35], 5% [20] 5.3% [20] 6% [21, 22, 34, 43], 7.5% [21, 40], and 10% [1, 21] and CP at concentrations of 10% [1, 2, 19, 22, 23, 30–33, 36–39, 42–46], 15% [34, 41, 46], 16% [23, 30, 38], 20% [46], and 28% [37]. A total of 8 studies analyzed the color change combining the use of spectrophotometer and visual color guides (classic vita pan type or similar) [1, 2, 19, 22, 23, 30, 36, 38], while 8 studies used color guides as the only recording method [20, 21, 33, 35, 39, 42, 44, 45]. One study used a spectrophotometer to determine color changes [37], 3 studies combined the use of colorimeter and color guides [40, 41, 46], and 4 studies used only a colorimeter [31, 32, 34, 43]. Some authors used customized splints adapted to the size of the sensor of the spectrophotometer or colorimeter, in order to favor the reproducibility of the measurements [1, 2, 19, 31, 32, 36, 40, 41]. Others simply placed the sensor in the center of the tooth, which is not as precise as the method described above [22, 23, 30, 37, 38, 43, 46].

**Table 1:** Summary of the methodology of the included studies

Authors	Year	Study type	Diagnostic method	Nº of participants (dropout)	Bleaching protocol	Application method	Age range	Bleaching agent
Meireles et al.	2022	RCT	Vita pan classical, Spectrophotometer	40(1)	G1:CP10% (Polanight 10%, SDI) 4 h weeks, preferably at night G2:CP10% (Polanight 10%, SDI) 4 h per day × 2 weeks, preferably at night	G1:ICWR G2:ICWR	> 18	CP10%
Martini et al.	2021	RCT	Spectrophotometer, Vita pan classical, Vita Bleach guide 3dmaster	46(10)	G1:PC10%(Opalescence, Ultradent) 3 h per day × 3 weeks G2:PC10%(Opalescence, Ultradent) 3 h per day × 3 weeks	G1:ICWR G2:ICWOR	NS	CP10%
Mailart et al.	2021	RCT	Spectrophotometer, Vita pan classical, Vita Bleach guide 3dmaster	60(0)	G1: HP10%(Opalescence go, Ultradent) 30 min per day × 2 weeks G2: HP10%(White Class) 30 min per day × 2 weeks G3: CP10% (opalescence)2 h per day × 2 weeks G4: PC10%(Opalescence) 8 h per day × 2 weeks	G1 ICWR G2 PCPM G3 ICWR G4:ICWR	> 18	CP10%, HP10%
Darriba et al.	2019	RCT	Spectrophotometer, Vita pan classical	50(0)	G1:CP10%(vivastyle vialdent ivoclar) 2 h per day × 2 weeks G2:CP10%(vivastyle vialdent ivoclar 2 h per day × 3 weeks	G1:ICWR G2:ICWOR	> 18	CP10%
Pinto et al.	2017	RCT	Vita Bleach guide 3dmaster	30(NI)	G1: HP6% (White Class with calcium – FGM)1 h per day × 7 days; G2, HP7.5%(White Class with calcium—FGM)1 per day × 7 days; G3 HP10% (Oral B 3D White – Oral-B) 30 min 2 times per day × 7 days G4 control group–placebo 1 h per day × 7 days	G1 ICWR G2: ICWOR G3: STRIPS G4: ICWOR	12–20	HP6%-7,5%,10%
Botelho et al.	2017	RCT	Colorimeter	26(2)	G1:CP15%(Nupro White GoldTM, Dentsply r) 2 h per day or all night × 3 months G2:HP6%(Crest Whitestrips 30 min 2 times per day × 3 months	G1: ICWR G2: STRIPS	287/304	CP15% HP6%
De Geus et al.	2017	CT	Vita clásico, Vita Bleach guide 3dmaster	60(17)	G1:Smokers, PC10%Opalescence) 3 h per day × 3 weeks G2 NONsmokers PC10%Opalescence) 3 h per day × 3 weeks	G1: PCPM G2: PCPM	18–54	CP10%
Aka et al.	2017	RCT	Vita pan classical, spectrophotometer	92(2)	G1:control G2:PC10% (Opalescence pf)8-10 h per day × 14 days; G3 HP6% (Opalescence Go) 1 h per day × 14 days;	G1 only color measurement G2: ICWR G3: PCPM	> 18	CP10% HP6%
Auschill et al.	2012	RCT	Vita pan classical	30(NI)	G1: HP5%(Colgate visible white) 30 min 2 v per day × 14 days G2: HP5,3%s (Whitestrips procter and gamble) 30 min per day 2 times per day × 14 days	G1: ICWR G2: STRIPS	18–56	HP5% HP5,3%
Turkun et al.	2010	RCT	Spectrophotometer	20(NI)	G1: CP28%(meta trayRemedent) 20 min per day × 10 days G2: PC10%(opalescence PF) 6-8 h per day × 10 days	G1:PCPM G2: ICWR	20–30	CP28% CP10%
Meireles et al.	2010	RCT	Spectrophotometer, Vita pan classical	92(11)	G1: CP10%(cp10) 2 h per day × 3 weeks G2: PC16% (Whiteness Perfect, FGM) 2 h per day × 3 weeks	G1:ICWR G2:ICWR	18–55	CP10%, CP16%
Meireles et al.	2009	RCT	Spectrophotometer, Vita pan classical	92(3)	G1: CP10%(cp10) 2 h per day × 3 weeks G2: PC16% (Whiteness Perfect, FGM) 2 h per day × 3 weeks	G1:ICWR G2:ICWR	18–55	CP10%, CP16%
Meireles et al.	2008	RCT	Spectrophotometer Vita pan classical	92(3)	G1: CP10%(cp10) 2 h per day × 3 weeks G2: PC16% (Whiteness Perfect, FGM) 2 h per day × 3 weeks	G1: ICWR G2: ICWR	18–55	CP10%, CP16%
Medeiros et al.	2008	CT	Vitapan classical	50(0)	G1: placebo G2:PC10% (Opalescence pf)8-10 h per day × 21 days;	G1: ICWR G2: ICWR	18–25	CP10%
Hannig et al.	2007	CT	Colorimeter	47(NI)	G1:HP6%(Whitestrips) 30 min per day, twice a day × 2 weeks G2:CP10%(vivastyle vialdent ivoclar)1 h per day × 2 weeks	G1: STRIPS G2: ICWR	18–60	CP10% HP6%
Matis et al.	2006	CT	Colorimeter Vitalescence Esthetic Restorative Masters Shade Guide	44(?)	G1: CP10% 6–8 h per day × 6 months G2: PC15% 6–8 h per day × 6 months G3: CP20% 6–8 h per day × 6 months SPLIT MOUTH DESIGN	G1: ICWR G2: ICWR G3: ICWR	> 18	CP10%, CP15%,CP20%
Leonard et al.	2003	CT	Vita pan classical	21(6)	G1: CP10% 6-8 h(Opalescence pf) per day × 6 months	G1: ICWR in a quadrant ICWOR on the other quadrant	> 18	CP10%
Myers et al.	2003	RCT	Vita pan classical	65(NI)	G1: HP3%(ADS Tooth Whitening Gel, Applied Dental Sciences, Inc., Lee, MA)) 30 min per day 3 times per day, tot 90 min) × 14 days G2: Placebo 30 min per day 3 times per day, tot 90 min) × 14 days	G1: ICWOR G2: ICWOR	> 21	HP3%
Matis et al.	2002	CT	Colorimeter, Vita pan classical	26(NI)	G1: CP15%(Rembrandt Xtra-Comfort Non- Sensitizing Bleaching Gel Regular Strength,)2hper day × 14 days	G1: ICWR on the left anterior quandrant ICWOR on the right anterior quandrant	23–68	CP15%
Mokhlish et al.	2000	CT	Trubyte bioform guide, Colorimeter	24(NI)	G1: CP20%(Opalescence pf))1 h 2 times per day × 14 days G2: HP7.5% (daywhite discuss dental) 1 h al 2 times per day × 14 days	G1: ICWOR G2: ICWOR	> 18	CP20% HP 7.5%
Swift et al.	1999	CT	Vita pan classical	29(5)	G1: CP10% 6-8 h per day × 2 weeks	G1: ICWOR	23–64	CP10%
Matis et al.	1998	RCT	Colorimeter	60(0)	G1: placebo 8–10 h per day × 21 days G2:PC10% (Opalescence pf) 8–10 h per day × 21 days	G1: ICWOR G2: ICWOR	+ 35–35	CP10%
Rosenstiel et al.	1996	RCT	Colorimeter	52(NI)	G1: CP10% 6–8 h(Opalescence pf) × 5 days G2: placebo 6–8 h × 5 days	G1: ICWOR	20–57	CP10%
Russel et al.	1996	RCT	Lumin Vacuum Shade Guide, Vita	50(NI)	G1: CP10% 6–8 h(Opalescence pf) × 2 weeks G2:placebo	G1: ICWOR	22–79	CP10%- placebo

*ICWR*, individualized cuvette with reservoir; *ICWOR*, individualized cuvette without reservoir; *PCPM*, preformed cuvette provided by the manufacturer; *RCT*, randomized clinical trial; *CT*, clinical trial; *CP*, carbamide peroxide, *HP*, hydrogen peroxide

**Table 2:** Summary of the results of the included studies

Authors	Year	Study type	Diagnostic method	Nº participants (dropout)	Results in terms of DSGU/ ΔE	Results: color change in ΔE values (spectrophotometer)	Results: color change in DSGU (shade guide units) values	Follow-up
Meireles et al.	2022	RCT	Vitapan Classical, Spectrophotometer	40(1)	DSGU + ΔE	G1:2 weeks 11.4(3.3)ª 1 month11.4(2.7)ª 3 months 10.5 (2.6)b G2: 2 weeks 7.6(2.4)ª 1 month7.1(2.3)ª 3 months 6.4(2.6)b	Used only in initial screening phases	3 months
Martini et al.	2021	RCT	Spectrophotometer, Vitapan Classical, Vita Bleach guide 3dmaster	46(10)	DSGU + ΔE	G1:baseline vs 1 month 8.4 ± 2.3 baseline vs 1 year: 9.0 ± 2.0 G2 baseline vs 1 month 18.6 ± 2.5 baseline vs 1 year: 8.9 ± 2.5	Vita classical: G1:baseline vs 1 month 8.1 ± 2.1 baseline vs 1 year: 8.0 ± 2.1 G2:baseline vs 1 month8.1 ± 1.8 baseline vs 1 year:8.1 ± 2.0 VITA Bleachedguide 3D-MASTER G1: baseline vs 1 month:10.4 ± 3.1 baseline vs 1 year: 9.2 ± 2.9 G2:baseline vs 1 month 10.8 ± 2.7 baseline vs 1 year 9.3 ± 2.7	1 year
Mailart et al.	2021	RCT	Spectrophotometer, Vitapan Classical, Vita Bleach guide 3dmaster	60(0)	DSGU + ΔE	G1: 2 weeks 7.03 ± 1.83 1 year 6.03 ± 2.33 G2: 2 weeks 6.75 ± 1.38 1 year 6.48 ± 1.02 G3:2 weeks 7.13 ± 2.75 1 year 5.67 ± 1.66 G4:2 weeks 7.49 ± 1.51 1 year 7.75 ± 2.04	Vita classica: G1: 2 weeks5.20 ± 0.99 1 year 5.30 ± 1.30 G2: 2 weeks 5.33 ± 1.85 1 year 4.61 ± 1.66 G3:2 weeks 4.63 ± 1.58 1 year 5.05 ± 1.93 G4:2 weeks 5.63 ± 0.95 1 year 5.76 ± 1.29 VITA Bleachedguide 3D-MASTER G1: 2 weeks6.70 ± 1.44 1 year 7.10 ± 1.05 G2: 2 weeks 6.86 ± 1.60 1 year 6.26 ± 1.42 G3:2 weeks 7.41 ± 1.40 1 year 7.05 ± 1.72 G4:2 weeks 7.70 ± 1.72 1 year 7.75 ± 1.62	1 year
Darriba et al.	2019	RCT	Spectrophotometer + Vitapan Classical	50(0)	DSGU + ΔE	G1:4.74 ± 1.94 4.81 ± 2.19 G2: 5.77 ± 2.15. 5.56 ± 2.09	G1: − 3.30 ± 3.12 -3.45 ± 4.04 G2: − 4.93 ± 3.91 − 5.15 ± 4.20	6 months
Pinto et al.	2017	RCT	Vita Bleach guide 3dmaster	30(NI)	DSGU	–	G1: 2M3/1 M G2: 3M1 /1M1 G3:3M1/1M1	1 year
Botelho et al.	2017	RCT	Colorimeter	26(2)	ΔE	G1:7.84 (3.88) 10.11 (3.42) G2:5.53 (4.16) 8.23 (4.69	–	3 months
De Geus et al.	2017	CT	Vita classical, Vita Bleach guide 3dmaster	60(17)	DSGU	–	VITA CLASSICAL G1:5.2 +—2.1. 4.7 +—2.2 G2:5.7 +—2.3 5.1 + -2.2 Vita 3d master G1: 4.1 +—1.1 2.6 +—1.1 G2:4.7 +—1.4 3.3 +—1.2	30 months (2.5 years)
Aka et al.	2017	RCT	Vitapan Classical, spectrophotometer	92(2)	DSGU + ΔE	G1:1.1,1,6- 1.0,1,3–1.0,1.3 G2:5.0,4.8- 7.0,6.9- 9.8,9.8 G3:3.3,3.3—4.8, 4.9- 5.7,6.2	NI	6 months
Auschill et al.	2012	RCT	Vitapan Classical	30(NI)	DSGU	G1;9.63 + -1.14 6.35 + -3.50 G2:9.68 + -0.95 6.41 +—2.65	–	18 months (1.5 years)
Turkun et al.	2010	RCT	Spectrophotometer	20(NI)	ΔE	G1:4.3 + -1.84 3.2 + -1.25 G2:9.3 +—2.4. 8.3 +—2.73	–	1 year
Meireles et al.	2010	RCT	Spectrophotometer, Vitapan Classical	92(11)	DSGU + ΔE	G1:8.5 (6.0–15.0) 2.9 (1.5–11.8) G2:8.8 (6.0–13.5) 2.7 (1.3–5.8)	NI	2 years
Meireles et al.	2009	RCT	Spectrophotometer, Vitapan Classical	92(3)	DSGU + ΔE	G1:8.5 (6.0 to 15.0) 2.7 (1.3 to 11.8) G2:8.8 (6.0 to 13.5). 2.7 (1.3 to 6.0)	G1:.8.7 (3.3 to 14.7). 2.5 (1.1 to 10.5) G2: 8.0 (4.3 to 12.7) 2.3 (1.3 to 5.7)	1 year
Meireles et al.	2008	RCT	Spectrophotometer, Vitapan Classical	92(3)	DSGU + ΔE	G1:8.5 (6.0 to 15.0) 3.0 (1.3 to 10.5) G2:8.8 (6.0 to 13.5). 2.4 (1.2 to 5.8)	G1:.8.7 (3.3 to 14.7). 22.5 (1.5 to 12.0) G2: 8.0 (4.3 to 12.7) 2.3 (1.0 to 6.2)	6 months
Medeiros et al.	2008	CT	Vitapan Classical	50(0)	DSGU	–	G1:2.04 2.04 G2:3.97 2.15	6 months
Hannig et al.	2007	CT	Colorimeter	46(NI)	Δl	G1: + 1.55 ± 0.41. 73.22 ± 1.59 G2:73.55 ± 1.83. + 1.20 ± 0.37		2 months
Matis et al.	2006	CT	Colorimeter Vitalescence Esthetic Restorative Masters Shade Guide,	44(?)	DSGU + ΔE	G1:9. /12 G2:9.5 /12 G3:11 / 12.5		5 years
Leonard et al.	2003	CT	Vitapan Classical	21(6)	DSGU	–	G1:C4(C1-C4) baseline B1(B1-C1) post treatment C1(A1-C3) a 90 months	90 months (7.5 years)
Myers et al.	2003	RCT	Vitapan Classical	65(NI)	DSGU	–	G1:7.0(D3) 12.0(A2) G2:7.0(D3) 8.0 (A3)	6 months
Matis et al.	2002	CT	Colorimeter, Vitapan Classical	26(NI)	DSGU + ΔE	ICRC:4.75 /3,5 ICSR: 3,75 / 3	NI	3 months
Mokhlish et al.	2000	CT	Trubyte bioform guide, Colorimeter	24(NI)	DSGU + ΔE	G1: CENTRAL:6.5/4 LATERAL: 6/3.75 CANINE:8/5 G2: CENTRAL:5/3.75 LATERAL: 5.5/3.5 CANINE:6.5/5.5		3 months
Swift et al.	1999	CT	Vitapan Classical	29(5)	DSGU	–	G1: D3 D2	2 years
Matis et al.	1998	RCT	Colorimeter	60(0)	ΔE	G1:1,8, 1,8, 1,5 / 1,8, 1,8, 1,5 G2:13.0, 9,6, 8.6 / 6,2, 4,9, 5,0	–	6 months
Rosenstiel et al.	1996	RCT	Colorimeter	52(NI)	ΔE	G1:4.6, 4,2 G2: 1.8,1.8	–	6 months
Russel et al.	1996	RCT	Lumin Vacuum Shade Guide, Vita	50(NI)	DSGU	–	G1: A3 A1 G2: D3 D3	6 months

The study with the lowest number of patients included 20 of them [37] and those with the highest number included 92 [22, 23, 30, 38]. Most of the studies opted for customized bleaching splints with a reservoir as the method of application of the bleaching agent [1, 2, 19–23, 30, 34, 36–38, 41–44, 46]. Six studies applied the agent in customized splints without a reservoir [31–33, 35, 39, 40]. Two studies applied the agent using customized splints with and without a reservoir, following a split-mouth design [

Regarding the bleaching agent application protocols, different patterns were observed. Most of the studies chose short exposure times between 30 min and 3 h [1, 19–21, 23, 30, 34–38, 40, 41, 43, 45]. The remaining studies opted for long exposure times between 6 and 8 h, maintaining a nocturnal pattern 

### Study results

Authors who measured color stability using objective techniques after 2 [43] and 3 months [2, 34, 40, 41] reported homogeneous results between groups. Slight recurrences were also described. However, they did not reach statistically significant values (*p* < 0.04). The studies that performed a 6-month follow-up [19, 22, 30–33, 35, 44] reported similar results. Authors who measured both ΔE and DSGU did not reach statistical significance ((*p* = 0.3, *p* = 0.7) [30] and (p > 0.05) [19]). Authors who only measured ΔE recorded stable values [31, 32]. The variations recorded indicated a slight regression of the color, but they were not statistically significant (*p* < 0.05). Three studies only performed DSGU measurements, two of them reported no recurrence [33, 35], and the third study [44] reported a setback of 1 DSGU unit and a recurrence of 12%.

Authors who performed follow-ups after 1 year post bleaching, did not report significant differences between the color change from immediate post bleaching and after 1 year. One study highlighted differences between beach guides, which were clinically significant after ΔE calculation [36]. Two studies [23, 38], in the annual and 2 years post-bleaching follow-ups, reported color stability in terms of the ΔE values.

In a study in which smokers were compared to non-smokers 2.5 years post bleaching [45], DSGU measurement showed a slight but statistically significant color recurrence in both groups. Two studies assessed patients with intrinsic discolorations secondary to the intake of tetracyclines [42, 46]. Follow-ups were performed at 5 and 7.5 years. The first study, ΔE results, showed that the result achieved after the treatment had maintained 68%, 67%, and 66% of the applied concentrations of CP (10%, 15%, and 20%, respectively) [46]. The second study, after 90 months, showed that 60% of the subjects reported no visible changes, 7% reported a slight recurrence, and another 7% showed a moderate recurrence [42].

### Risk of bias

**Fig. 2:**
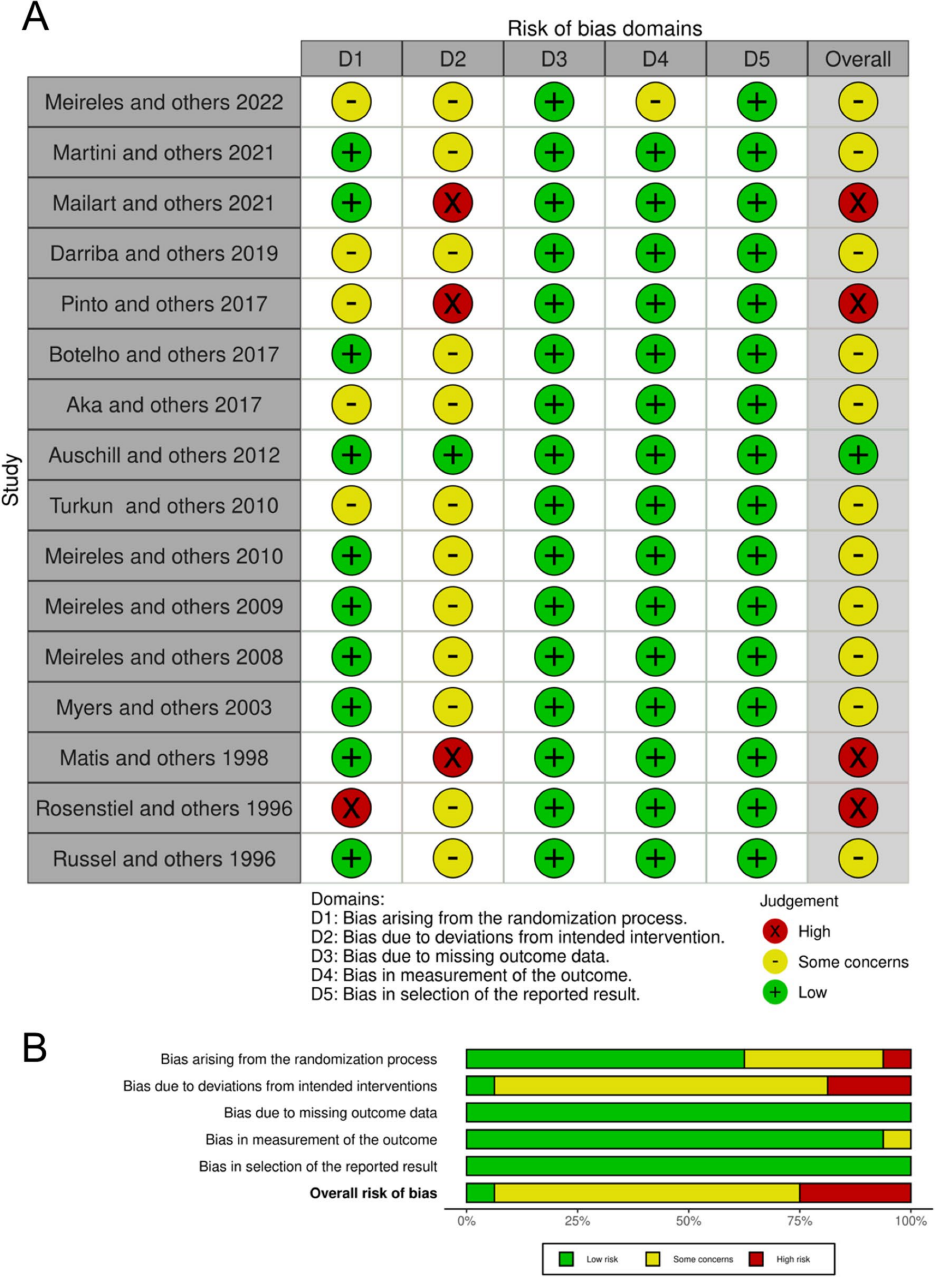
Quality assessment of the included RCTs using RoB-2. **A** Table format. **B** Graphical format

**Fig. 3:**
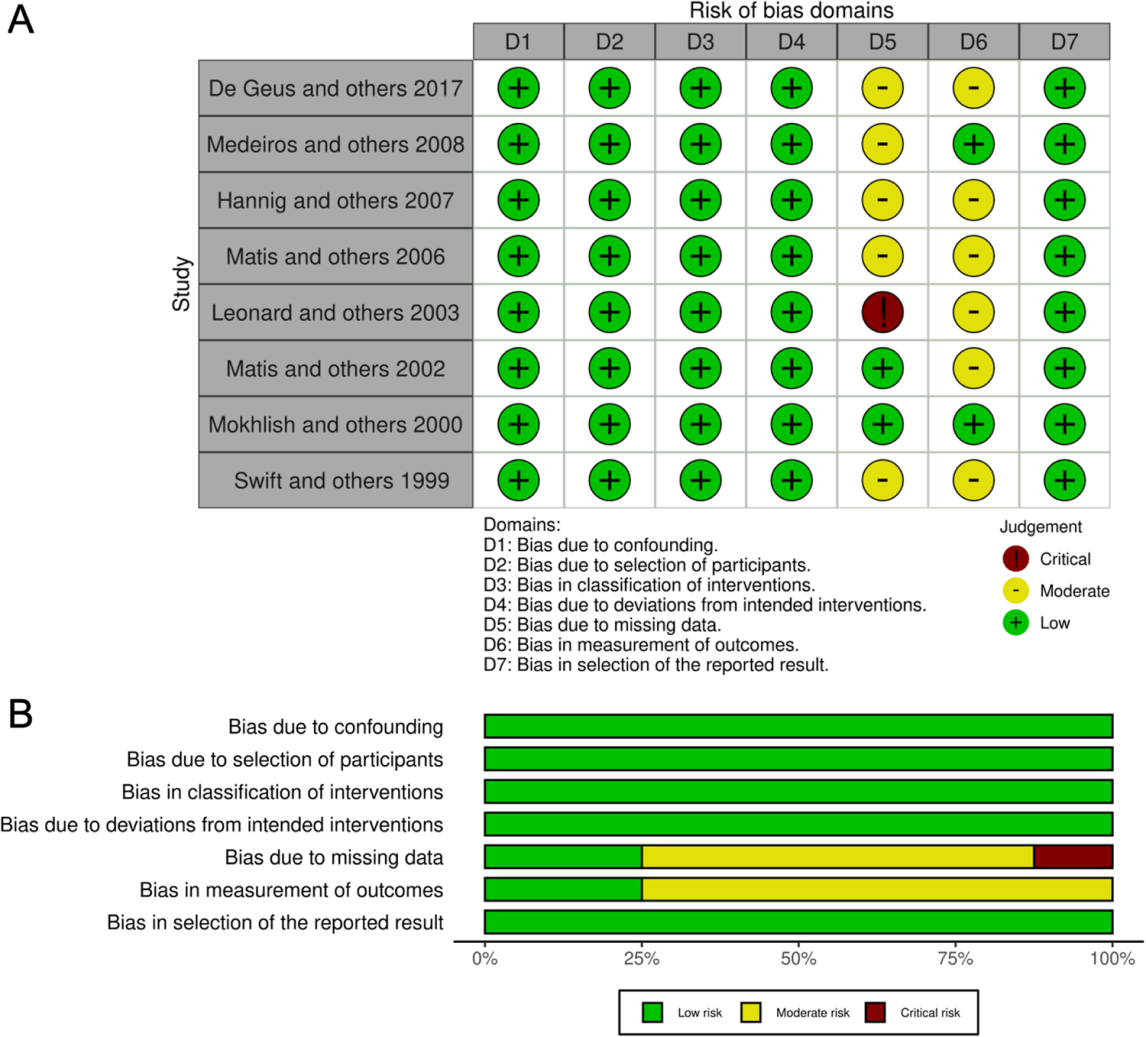
Quality assessment of the included CTs using ROBINS-I. **A** Table format. **B** Graphical format

All studies were rated using the Cochrane Collaboration tools for risk of bias: RoB2 was used for rating RCT and ROBINS-I for CT (Figs. 2 and 3).

RCT studies were classified as unclear for the following elements: bias arising from the randomization process [2, 19, 21, 22, 37], bias due to deviations from the intended intervention [2, 19, 22, 23, 30, 32–38], and bias in outcome measurement [2]. Other studies were classified as high risk for the following elements analyzed: bias arising from the randomization process [32] and bias due to deviations from the intended intervention [1, 21, 31]. The remaining studies were classified as low risk for the following elements: bias arising from the randomization process [1, 20, 23, 30, 31, 33–36, 38], bias due to deviations from the intended intervention [20], bias due to missing data on outcomes [1, 2, 19–23, 30–38], bias in outcome measurement [1, 19–23, 33–41], and bias in selection of the reported outcome [1, 2, 19–23, 30–38].

CT studies were classified as low risk for the following elements: bias due to confounding [39–46], bias due to the selection of participants [39–46], bias in classification of interventions [39–46], bias due to deviations from intended interventions [39–46], bias due to missing data [40, 41], bias in measurement of outcomes [40, 44], and bias in the selection of the reported result [39–46]. Other studies were classified as unclear for the following elements: bias due to missing data [39, 43–46] and bias in measurement of outcomes [39, 41–43, 45, 46]. Only one study was classified as high risk for the following element: bias due to missing data [42].

## Discussion

Concern has been expressed about the duration of the effects of dental bleaching. This has been the subject of several studies over the years [3, 4]. Multiple variables such as effectiveness, intra- and post-treatment sensitivity, and long-term color stability have been analyzed [47]. The great heterogeneity in the designs of the research methodologies, the lack of a single clinical and follow-up protocol (or at least a minimum for conducting studies), and the different techniques applied to obtain the data make it difficult to reach a consensus. Nevertheless, the results obtained in the included studies highlight that the color obtained after home dental bleaching remains stable between 1 and 2.5 years regardless of the bleaching agent used or its forms of administration.

The selected studies compared the performance of at-home bleaching products by applying different experimental protocols with the aim of monitoring their long-term prognosis. The number of participants was different among the studies, ranging from 20 to 92. The remaining articles included an average of 51 participants, and only a few studies performed a sample size calculation [1, 19, 22, 23, 30, 36, 38].

The studies included variables such as the presence or absence of a reservoir in the bleaching splints to determine if its presence influenced the result [41, 42]. Color measurements were performed by objective technique, such as the spectrophotometer [37] or colorimeter [31, 32, 34, 43], or subjective operator dependent techniques, using shade guides sorted according to dental brightness [20, 21, 33, 35, 39, 42, 44, 45]. Other studies used a combination of both techniques [1, 2, 19, 22, 23, 30, 36, 38, 40, 41, 46].

The analysis and comparison of the data obtained from the different studies present some limitations since the authors performed the measurements not only in different ways, but also at different times and with different apparatus. In addition, when the measurements were performed with the same apparatus (spectrophotometer or colorimeter), they were not carried out in the same way. In the case of measurements using subjective techniques, it should be noted that the same shade guides were not used in all the studies. This hinders the comparability between studies.

The clinical and follow-up protocols were diverse. The results obtained in the studies regarding the effectiveness of the method of application of the bleaching agent were similar. Therefore, it can be deduced that the form of application of the treatment does not influence the final result. The extent of treatment over time varied between 1 and 3 weeks. In the case of intrinsic staining treatments secondary to drugs (tetracyclines), the treatment was extended up to 6 months [42, 46]. Follow-up times were also heterogenous, ranging from 2 months to 7.5 years.

Authors who measured color stability using objective techniques after 2 [43] and 3 months [2, 34, 40, 41] and who used HP between 6% [34, 43] and 7.5% [40] and CP between 10% [2, 41, 43], 15% [34, 41], and 20% [40] reported homogeneous results between groups. Slight recurrences were also described. However, they did not reach statistically significant values (*p* < 0.04). Such results could be attributed to the short follow-up time.

The studies that performed 6-month follow-up and applied CP from 10% [19, 22, 30–33, 44] to 16% [30] and HP at 3% [35] and 6% [22] and performed the measurements subjectively [33, 35, 44], objectively [31, 32], and by means of a combination of both [19, 22, 30] and reported similar results. Rosentisel et al. [32] and Matis et al. [31] measured color using a colorimeter. Both applied 10% CP overnight but differed in the extent of treatment: 5 days and 3 weeks, respectively. They agree that after the color measurements and subsequent CIELAB analysis calculating ΔE, the color was stable when compared to the post-bleaching values. Also, the variations recorded indicated a slight regression of the color, but they were not statistically significant (*p* < 0.05).

In the studies by Meireles et al. [30] and Darriba et al. [19], in which color measurements were made using both techniques, despite having applied different bleaching protocols, the authors reported a slight recurrence in both groups studied. However, again, data did not reach statistical significance ((*p* = 0.3, *p* = 0.7) [30] (*p* > 0.05) [19]). Color measurement by means of color guides confirmed the results obtained after calculation of ΔE value. On the other hand, Aka et al. [22] observed a statistically significant (*p* < 0.05) recurrence (ΔE) in the CP group at 10%, and the measurement of color using guides showed better brightness values in the CP group compared to the HP group.

Alternatively, Russell et al. [33], Mayers et al. [35], and Medeiros et al. [44] performed the measurements only by using shade guides. The first two reported no recurrence, while Medeiros et al. [44] reported a setback of 1 DSGU unit and a recurrence of 12%, without attributing this result to a specific cause. The subjectivity of the technique and the difficult comparability of the values derived from it with the values provided by objective techniques, which are capable of recording what the human eye cannot always record, or at least not with such precision, make it difficult to compare the studies with each other.

Other authors performed follow-ups after 1 year post bleaching. Martini et al. [36] performed color measurements using a spectrophotometer and color guides. They reported no significant differences between the color change from immediate post bleaching and after 1 year using the classic vita pan shade guide unit (mean difference = 0.1 ΔSGU; 95% central incisor, 0.2 to 0.4; *p* = 0.53; and at 1 year, mean difference = 0.3 95% central incisor 1.0 to 1.6; *p* = 0. 62). However, significant differences were observed using the VITA Bleachedguide (VITA Zahnfabrik, Bad Säckingen, Germany) (mean difference = 1.4 ΔSGU; 95% central incisor 0.7 to 2.1; *p* < 0.01). After calculation of ΔE, these differences were defined as not clinically important, since they were below the 50:50 perception threshold for shade changes in dentistry [36].

Another factor that hinders the comparison of the results of studies using subjective techniques is the diversity between guides. This generates a certain degree of uncertainty about the reliability of the results obtained. Authors such as Mailart et al. [1] and Martini et al. [36] performed follow-up at 1 year using a spectrophotometer and two different color guides and concluded that the color remains stable since no statistically significant differences were observed between the groups after ΔE analysis. In addition, no color discrepancies were reported upon comparison with the two guides. Meireles et al. [23, 38] also performed the color measurements using a spectrophotometer and color guide. In the annual and biennial post-bleaching follow-ups, the results were not significantly different in terms of the ΔE values, and the median tooth color did not show statistically significant changes (*p* > 0.2).

Pinto et al. [21] and Auschill et al. [20] analyzed the shade stability using vita 3DMASTER (VITA Zahnfabrik, Bad Säckingen, Germany) and vita pan classic (VITA Zahnfabrik, Bad Säckingen, Germany) shade guides, respectively. In the study by Pinto et al. [21], all groups showed color stability at 12 months post bleaching. Auschill et al. [20], after 18 months of follow-up, reported recurrence in both groups studied when compared with the results obtained after bleaching, reporting values from 2.88 (*p* = 0.24) to 3.3 (*p* = 0.001) DSGU.

The origin of discolorations will be decisive in determining both the effect and the prognosis of bleaching. The study conducted by De Geus et al. [45] selected a different sample from the previously mentioned studies, composed of smokers vs. nonsmokers. The aim of their study was to evaluate the stability of whitening at 2.5 years using measurement techniques using classic vita pan and vita bleach 3dmaster shade guides (VITA Zahnfabrik, Bad Säckingen, Germany). At 30 months, a homogeneous high dropout rate (28%) was reported in both groups: 5 participants stopped smoking and 4 reduced the number of cigarettes consumed daily. In the measurements, a slight but statistically significant color recurrence could be detected in both groups. Therefore, the recurrence is not only attributable to extrinsic staining potentially caused by smoking, although in view of the changes in the smoking group, the comparison is not completely fair. The authors whose samples consisted of patients with intrinsic discolorations secondary to the intake of drugs (tetracyclines) were Matis et al. [46] and Leonard et al. [42], who applied similar clinical protocols: nocturnal application of CP for 6 months, at 10%, 15%, and 20% in the case of Matis et al. [46] and only 10% in the case of Leonard et al. [42]. The color measurement techniques and follow-up times varied between the two studies. In the first study [46], they followed up at 5 years using a colorimeter and Vitalescence Esthetic Ultradent shade guides (Vitalescence Esthetic Restorative Masters Shade Guide, Ultradent Products, Inc.), and in the second study [42], they followed up at 7.5 years measuring shade changes using classic vita pan shade guides. Matis et al. [46] reported ΔE results, which showed that concentrations of 10%, 15%, and 20% had maintained 68%, 67%, and 66% of the color achieved after bleaching, respectively. The results of Leonard et al. [42] also showed recurrences. After 90 months, 60% of the subjects reported no visible changes, 7% reported a slight recurrence, and another 7% showed a moderate recurrence, but never matching the pretreatment color. In addition, 27% of the subjects reported having undergone further bleaching sessions before 90 months. Both studies showed higher levels of recurrence than studies whose samples did not include patients with intrinsic staining, which is evidence of a greater degree of difficulty in maintaining the results in these groups of patients.

The main limitation of the present systematic review is based on the great diversity of clinical and follow-up protocols employed in the included studies. In addition, a wide range of measurement methods were used, which make it difficult to compare the results obtained in the studies. Therefore, there is a need to establish a single experimental protocol to facilitate the interpretation of the data obtained.

## Conclusions

Although many studies have demonstrated the general efficacy of bleaching gels, the long-term benefits of the treatment related to color maintenance are not well established in the literature. Most authors state that the color remains stable between 1 and 2.5 years regardless of the type of bleaching agent or the forms of administration such as individualized cuvette with reservoir, individualized cuvette without reservoir, and preformed cuvette provided by the manufacturer. Color stability in cases of severe discolorations such as tetracycline staining presents a higher degree of recurrence.

## Supplementary Information

Below is the link to the electronic supplementary material.

Supplementary file1 (DOCX 16 KB)
