# Supplementary material for: Prognosis in home dental bleaching: a systematic review
Source: Clin Oral Investig. 2023 Jun 5;27(7):3347–61. doi: 10.1007/s00784-023-05069-0 (PMC10329590; doi:10.1007/s00784-023-05069-0)
Supplement: Supplementary file 1 — Supplementary file1 (DOCX 16 KB) [file 784_2023_5069_MOESM1_ESM.docx]

**Supplementary Table 1.** Search strategy

| Database | Search strategy | Findings |
| --- | --- | --- |
| Pubmed | **#1** ((prognosis[MeSH Terms]) OR (prognosis)) OR (dura*) | 2,939,870 |
|  | **#2** (((((((agents, tooth bleaching[MeSH Terms]) OR (agents, tooth bleaching[MeSH Terms])) OR (bleaching agents[MeSH Terms])) OR (agents, teeth whitening[MeSH Terms])) OR (agents, tooth whitening[MeSH Terms])) OR (agents, whitening[MeSH Terms])) OR ("tooth bleaching" or "teeth bleaching" or bleaching)) OR ("tooth whitening" or "teeth whitening" or whitening) | 15,599 |
|  | **#3** home | 315,036 |
|  | **#1 AND #2 AND #3** | **138** |
| Web of Science | **#1** ALL FIELDS (prognosis or dura*) | 1,557,040 |
|  | **#2** ALL FIELDS ("tooth bleaching" or "teeth bleaching" or "dental bleaching" or "dental whitening" or bleaching or whitening) | 32,420 |
|  | **#3** ALL FIELDS (home) | 345,144 |
|  | **#1 AND #2 AND #3** | **33** |
| Embase | **#1** ALL FIELDS (prognosis or dura*) | 2,860,949 |
|  | **#2** ALL FIELDS ("tooth bleaching" or "teeth bleaching" or "dental bleaching" or "dental whitening" or bleaching or whitening) | 276,855 |
|  | **#3** ALL FIELDS (home) | 860,309 |
|  | **#1 AND #2 AND #3** | **54** |
